# An Attack on the Almoner System

**Published:** 1913-05-24

**Authors:** 


### AN ATTACK ON THE ALMONER SYSTEM.

### Adjourned Meeting of the Hospital Saturday Fund.

The adjourned meeting of the Board of Delegates of the Hospital Saturday Fund was held on Saturday, the 17th inst., with Mr. Harry Gower, chairman of the Council, in the chair, to consider the report of a Special Committee on Inquiries at Hospitals under the Almoner System, which has been enforced upon the Metropolitan hospitals by the King's Fund. This system is established to check abuse by patients, and to provide that none but suitable applicants shall receive free treatment at these institutions. We published the substance of this report in The Hospital of April 12, 1913, page 32. Its main recommendation was to the effect that all patients sent to the hospitals by the Saturday Fund should be exempted from the almoner's inquiries, and should be medically treated without such investigations. The committee considered that all general hospitals should issue leaflets giving particulars of attendance and other information essential for the guidance of applicants for treatment.

We are indebted to the *Daily Telegraph* for the following report of the proceedings :—

After discussion (lasting two hours) it was resolved :

(*a*) "That each patient for a hospital at which the almoner system is in operation be furnished with a letter of recommendation from the Fund."

(*b*) "That all such hospitals which have been awarded grants by the Fund be requested to accept and honour such recommendations without further inquiry."

(*c*) "That it be an instruction to the distribution committee to report to the Board the names of participating hospitals which decline to agree to accept such recommendations without inquiry, and on that report it shall be in order for the Board to pass a resolution declining to award further grants to such hospitals."

The committee considered that all general hospitals should issue leaflets giving particulars of attendance and other information essential for the guidance of applicants for treatment.

Mr. J. Neal (hon. secretary) expressed the opinion that the Fund had lost more by the almoner system than they had through the operations of the National Insurance Act.

The Secretary (Mr. A. W. Davis) stated that the flood of complaints which reached the offices of the Fund in the latter part of January last ceased as suddenly as it began. Subscribers had then not chosen their insurance doctors, and had no knowledge that their ordinary ailments would in future have to be treated by insurance doctors. Since February 1 complaints against hospitals had almost entirely ceased, the number being considerably below the average for this time of the year. All complaints that had been referred to hospitals had been fairly and fully inquired into; where errors of judgment had been made remedies were applied, and where it was possible to give further consideration or grant concessions the hospitals did their utmost to meet the wishes of the Fund.

### The View of the Hospitals.

A letter was read from Mr. E. W. Morris, Secretary of the London Hospital, dated 9th ultimo, in which he reported how the proposal of the sub-committee *re* the almoners, and the attitude adopted by some of the speakers at the delegates' meeting, would be regarded by the hospitals themselves. Mr. Morris's points may be summarised as follows :—

(1) The hospitals have always regarded the grants of the Saturday Fund as gifts charitably given; they do not consider such grants as a contribution to a provident dispensary scheme, as some contributors to the Saturday Fund deem. If the latter contentions embody the views of the majority of the delegates, the hospitals should be immediately informed of the fact, when Mr. Morris feels that none of them would accept a grant on such terms. Mr. Morris feels convinced that no hospital would undertake the treatment of a body of men on any basis of insurance.

**(2) The resolution now passed, as set forth above, in effect presents a pistol "at the hospitals' heads," which would seal the doom of the Saturday Fund.** Mr. Morris ascertained from the secretaries throughout London that, without a single exception, their hospital would not accept any grant from the Hospital Saturday Fund on any such conditions. Indeed, the Saturday Fund might run the risk of getting some retaliation, for the hospitals who had refused to accept the grants of the Saturday Fund might direct subscribers to the Saturday Fund who appealed to the hospital for help to attend such hospitals as had accepted those conditions. The Saturday Fund might, therefore, run the risk of being deprived of the services of all the larger general hospitals.

Mr. Morris further expressed regret that the Hospital Saturday speakers should have so very unfortunate an opinion of the habits of lady almoners; he is sure that such practices as were described by the delegates are not carried out at the London nor at any of the larger hospitals. The almoners always speak of the Saturday Fund as one of their best friends and helpers.

### Mr. Morris's Four Serious Objections.

Mr. Morris urged the following serious objections to the proposal of the Saturday Fund :—

(*a*) The contributors to this Fund ought to see that the almoners are intended to protect all contributors, of which the Saturday Fund is one. If the wishes of some of the speakers were carried out and all inquiry at the hospitals was abolished, the income of the hospital would be wasted in treating the cases which on no account ought to receive gratuitous treatment, and there would be little to spend on necessitous cases.

(*b*) The resolution as passed puts the hospitals, and, indeed, the Saturday Fund itself, in this strange position—namely, that one fund (King Edward's Hospital Fund) recommends the extension of the almoner system, whilst the Saturday Fund recommends its restriction.

(*c*) Some speakers wrongly think that patients visited the Inquiry Office, and that their treatment was cancelled before they had seen the doctor. This is never the case at any hospital where almoners are employed, and the treatment of a patient is never cancelled by the almoner until after such patient has seen the doctor, and then only when the doctor himself agrees. In the Casualty Department at the London there are no almoners, and the doctors themselves decide whether the patient's treatment shall be continued or not.

(*d*) The various hospitals have their own individual rules. For instance, some hospitals have a wage-limit, others have not; some treat one kind of case which others refuse—syphilis, for instance; some treat insured persons, whilst others do not; some will give material help to unmarried women, others will not. Therefore, an almoner, in deciding whether any patient should be treated or not, has to remember what are the rules of her own hospital with regard to such a patient. How could such hospitals possibly agree to treat any case sent to them by an outside person who can know nothing of their rules?

### General Objections.

Mr. Morris rightly contends that if the Saturday Fund wishes to refuse the attention of the almoners when applied critically, they must also deprive themselves of the benefit of the almoners when used helpfully, which is a much more important part of the almoner's work.

The almoner, for instance, obtains admission to convalescent homes, extra food for out-patients, and arranges for the home visitation of nurses. The almoners further help in getting employment by the system of apprenticeship, etc. Now the Saturday Fund wish to secure for their subscribers all this help, and yet they demand that every hospital shall agree that, whilst the almoners are to grant these benefits to a certain class, they are to make no inquiries as to the suitability of these very people to receive the benefits in question.

### The Only Real Question at Issue.

The whole question at issue turns on one point, and one only. Is the grant from the Saturday Fund to the hospitals a payment for services rendered, or to be rendered, or a gift? If a gift, the hospitals will continue to protect the giver, as they protect all other givers. If a payment, the hospitals will either refuse it altogether, or else reply that if there is any question of payment then the payment must be to some extent equal to the benefits given, and while such benefit is less than 1 per cent. of the expenditure of the hospital in its work, the Saturday Fund is not in a position to take up that attitude.

**The Hospital Saturday Fund's total contributions to the general and special hospitals of London do not exceed $3\frac{1}{2}$ per cent. of their ordinary income.**

We have dealt with this matter in a separate article on another page of this issue.

